# Social Stability Risk Assessment of Disaster-Preventive Migration in Ethnic Minority Areas of Southwest China

**DOI:** 10.3390/ijerph19106192

**Published:** 2022-05-19

**Authors:** Linyi Zhou, Demi Zhu, Wei Shen

**Affiliations:** 1 School of International and Public Affairs, Shanghai Jiao Tong University, Shanghai 200030, China; zhoulinyi1017@sjtu.edu.cn (L.Z.); zhudemi@sjtu.edu.cn (D.Z.); 2 School of Economics and Management, Tongji University, Shanghai 200092, China

**Keywords:** disaster-preventive migration (DPM), social stability risk, fuzzy comprehensive evaluation (FCE), ethnic minority area, China

## Abstract

Disaster-preventive migration (DPM) is an important method for disaster risk management, but migration itself entails a potential social stability risk. This study took County D in Yunnan Province, one of the counties most severely threatened by geological disasters in China, as an example to construct an indicator system of social stability risk factors for disaster-preventive migration based on a literature survey and in-depth interviews. The system consists of 5 first-level risk factors and 14 s-level risk factors. The social stability risk of DPM in County D was assessed using a fuzzy comprehensive evaluation method based on experts’ weights. The results showed that the overall social stability risk level of disaster-preventive migration in County D is ‘high’. In terms of importance, the five first-level risk factors were ranked as follows: public opinion risk > compensation risk > livelihood recovery risk > cultural risk > geological disaster risk. Among the risk factors, the level of public opinion risk and compensation risk appeared to be high, whereas that of livelihood recovery risk, cultural risk and geological disaster risk resulted to be medium. To our knowledge, this paper is the first research to evaluate the social stability risk of DPM; it not only enriches the theories of social stability risk assessment, but also has important guiding significance for people relocation and resettlement in Chinese ethnic minority areas.

## 1. Introduction

According to the Global Risks Report 2020, natural disasters rank third in terms of likelihood and seventh in terms of impact, among all potential global risks [1]. From 1975 to 2015, the number of people exposed to risks associated with all types of natural disasters has doubled globally as a result of urbanization, population growth, and socioeconomic development [2]. Natural disasters pose a greater risk to and have a more devastating impact on less developed countries or regions, and poor people are the most vulnerable to their effects [3]. China is particularly prone to natural disasters [4]. In 2020, 138 million people were affected by various natural disasters in China; furthermore, 591 people died or went missing due to such disasters, while the direct economic losses are estimated to be 370.15 billion yuan [5]. Geological disasters are natural disasters caused by a geological process and include earthquakes, volcanic eruptions, landslide, mud-rock flow, etc. [6]. Compared with the eastern and central regions, western China is ecologically sensitive and more susceptible to natural disasters; these areas also have a lower capacity to avert the risk of natural disasters, which means that a natural disaster has severe consequences in these regions. For example, a magnitude-6.4 quake hit Yunnan province in southwest China at 9.48 p.m. on Friday 21 May 2021 local time, and just hours later, a magnitude 7.4 earthquake hit the Qinghai Province in northwest China. The two massive earthquakes killed at least three people and injured dozens of others.

Migration is one of the most effective methods by which human beings respond to recurrent natural disasters [7]. When people are unable to adapt to or mitigate the impact of natural disasters in an area prone to such disasters, there is no alternative to migration [8]. To minimize the risk of natural disasters, the Chinese central government formulated the Decisions on Strengthening the Prevention and Control of Geological Disasters, which emphasized accelerating the relocation and evacuation of people living within areas particularly vulnerable to geological disasters. The document suggested that this process should be accelerated through a series of planned steps, and that priority must be given to the relocation of people living around potential spots of geological disasters that were highly hazardous or difficult to manage [9]. After more than a decade of development, the Chinese government recently implemented a relocation and resettlement plan in parallel with the risk identification and management of natural disasters [10].

However, there are several potential risks associated with disaster-preventive migration. Downing identified nine major risks of resettlement, namely, joblessness, homelessness, marginalization, food insecurity, loss of common land and resources, increased health risks, social disarticulation, disruption of formal educational activities, and loss of civil rights [11]. Migration may also lead to a decline in the productivity and standard of living of people compared with their lifestyle in their place of origin, disrupt their customs and the living environment, as well as create ongoing conflicts between the affected population and the local authorities when government compensation falls short of expectations [12,13,14,15]. In some cases, this situation may give rise to violent resistance and bloody conflicts, thereby posing a threat to the harmony and stability of the society [16].

In this context, relocation in County D of Yunnan Province, China, is a much more complicated issue compared with regular relocation—the county is multi-ethnic, is located at a high altitude, and is prone to natural disasters. Disaster-preventive migration in this county has threatened social stability and therefore has faced significant resistance. This leads to a research question: how to identify and evaluate the social stability risk of DPM in County D? A solution to this problem would not only promote the development of social stability risk assessment but may also serve as an important guide for the relocation and resettlement of DPM populations belonging to minority groups.

Concretely speaking, this paper attempts to answer the following two fundamental issues:

(1) What are the core risk factors for social stability associated with disaster-preventive migration in county D?
(2) How to use scientific methods to assess the social stability risk?

## 2. Literature Review

Natural disasters are closely associated with property damage and migration [17,18]. Disaster-preventive migration is a risk management strategy for relocating and resettling people exposed to the risks of natural hazards [19]. Disaster-preventive migration, which is different from migration for specific projects, ecological restoration, and natural resource conservation, aims to protect the property and lives of populations exposed to natural disasters and may cause negative impacts on the resettled population [20,21]. While resettlement may result in better housing quality for people, their economic well-being may be negatively affected [22]. In addition, some people who relocate may not be able to adapt to the new environment, leading to a deterioration in their mental health status [23] and a decrease in subjective well-being [24]. As a result, disaster-preventive migration is often used as the last option for the prevention of risks linked to natural disasters [25].

Disaster-induced migration is divided into post-disaster migration and disaster-preventive migration. Post-disaster migration takes place when it is not possible for residents to return to the original settlement after a disaster [19]. Disaster-preventive migration is considered a possible strategy to cope with disaster risks linked with the increased possibility of natural disasters [26,27]. Some countries have begun to include disaster-preventive migration as an important component of their national disaster management strategies [28]. However, even when people’s means of living are threatened by natural disasters, sentimental attachment to the local residence is an important reason for their reluctance to relocate [29,30]. Therefore, distant and permanent resettlement as part of disaster-preventive migration is an important cause of social instability [31,32].

To prevent projects’ failure, international organizations and the national migration planning authorities of numerous countries require an ex ante assessment of the potential harmful consequences of migration projects [33]. Social impact assessment (SIA) is a method commonly adopted internationally for managing the social issues related to projects [34]. SIA originated from the environmental impact assessment (EIA) [35] framework in the United States and has now become a complete assessment system over the course of 50 years [33,36,37]. The application of SIA around the world has contributed significantly to the success of many projects. However, due to policy and institutional issues, SIA has faced many obstacles during the implementation stage in developing countries [38] such as China [39]. Social stability risk assessment is a nascent system that respects Chinese particularities and was developed based on SIA. Although the institutional framework for SSRA in China has been refined thereafter, current studies have focused on the construction of large engineering projects [40,41,42], meteorological disasters [43], land acquisition [33], and migration engineering [44], among other issues. There is a lack of research on the SSRA of preventive migration in areas at high risk of experiencing geological disasters.

It is undeniable that social stability risk assessment is a tool for planning better resettlement [34]. The social stability risk linked to DPM comes from various sources including livelihood recovery, land compensation, cultural factors, and so on [45,46]. The immigrant livelihood issue is a topic of common concern for scientific research and policy debates [47]. As livelihood recovery is the key to sustainable development in disaster-affected areas [48], current research has focused on livelihood asset assessment [49,50,51], livelihood vulnerability assessment [52,53], and livelihood risk mitigation strategies [54,55]. Land compensation policies are directly related to the livelihood of immigration, especially of farmers, and have a major influence on disaster-related migration aspirations [56]. Cultural factors play both positive and negative roles in natural disaster risk management. Religion is considered to be a part of culture and communities and plays an important role in natural disaster management because it has access to resources that are vital to emergency rescue in a disaster [57]. However, some believers misinterpret natural disasters, for example, earthquake and tsunami in Japan are regarded as God’s warning, and this has a negative impact on natural disaster management [58].

Involuntary migration disrupts people’s routine life and causes them to become uncomfortable. Studies have shown that involuntary migration can be extremely detrimental to the social stability of the relocated communities [59]. Most residents, living in areas identified as particularly prone to natural disasters, are reluctant to leave their homes [60] because of place attachment [61,62], loverhood, and religion [63,64,65]. Improving the risk perception of natural disasters is an important measure to promote relocation decisions [66]. Risk perception is a subjective judgment that people make about the characteristics and severity of a risk [67]. A high natural disaster risk perception is reflected in the purchasing of a natural disaster insurance [68], the enhancement of DPM willingness, and the adoption of a natural disaster risk reduction strategy [69,70].

The possible marginal contributions of this paper include: (1) risk perceptions was incorporated into the index system of SSRA, thus improving the SSRA framework; (2) this study focuses on the SSRA of geological disaster migrants, which may broaden the research field of SSRA; (3) in terms of evaluation method, we established a fuzzy comprehensive evaluation method considering multi-experts’ weights to assess the social stability risk of DPM; the method not only considers the ambiguity of the evaluation problem, but also solves the inconsistent problem of experts’ views.

## 3. Materials and Methods

### 3.1. Study Area

**Figure 1:**
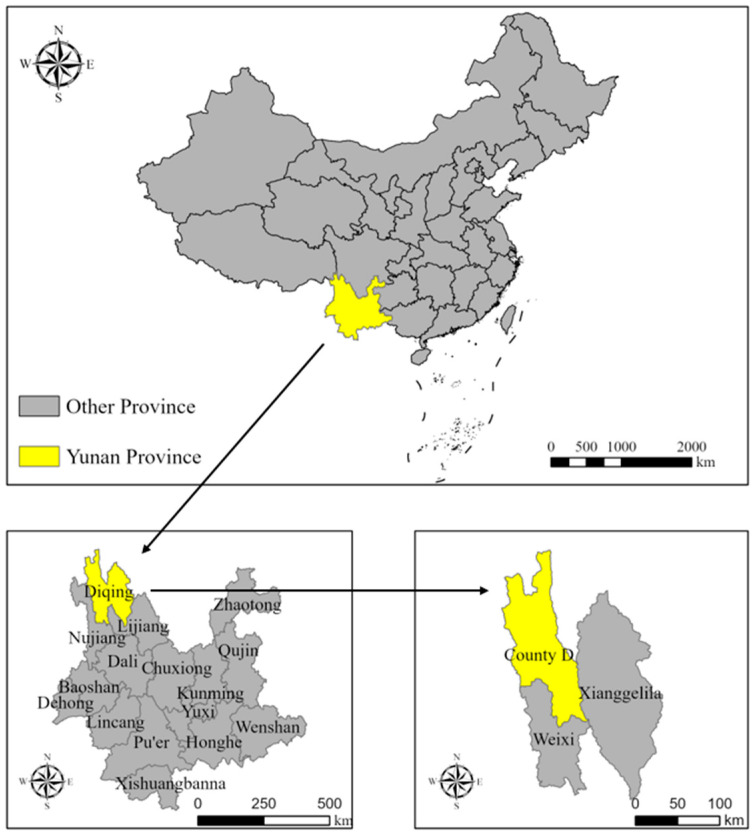
Study area.

County D, in Diqing Tibetan Autonomous Prefecture in Yunnan Province, is located between 98°3′56″–99°32′20″ E and 27°33′44″–29°15′2″ N in the Hengduan mountain range in the northwestern part of Yunnan Province. There are two towns and six townships in County D, with a total area of 7291 square kilometers and a population density of eight people per square kilometer. S Town, the county capital, is located at 3400 m above sea level; it is 182 km from Shangri-La, the state capital, and 889 km from Kunming, the provincial capital. Since ancient times, S town has presented an intersection of Chinese and Tibetan cultures, served as the ‘foreign market center’ along the southwestern Tea Horse Road, and operated as the Tibetan-Chinese trade and cultural distribution center. It is known locally as the ‘snow mountain foreign market’. S town is an important transportation hub from Yunnan to Sichuan and Tibet and occupies a special strategic location for political, economic, and cultural communication between Yunnan, Sichuan, and Tibet. It is also the political, economic, and cultural center of County D. S town is one of the main settlements of ethnic minorities. Major ethnic groups include the Tibetan, Han, Hui, and Naxi. It is also a typical multi-religious area, including Tibetan Buddhism, Catholicism, and Islam, among others. Due to a complex geological structure, this area is prone to geological disasters such as landslides and mudslides and it is one of the key target areas for geological disaster prevention and control [71]. The disaster-preventive migration in focus involved two communities and one village in S town of County D, comprising more than 1700 households and approximately 6000 people (Figure 1).

### 3.2. Data Sources

The data in this study were derived from on-site expert surveys, symposiums, and face-to-face interviews. In July 2020, a survey team was selected by the People’s Government of County D and was divided into five groups to investigate the social stability risks from disaster-preventive migration in County D. First, symposiums for experts were held for government departments and scholars in the field of migration in County D. Questionnaires were distributed to the experts who took part in the symposiums, to collect data. These experts belonged to the People’s Government office of County D, the Development and Reform Commission, the Planning and Land Bureau, the Housing and Urban-Rural Development Bureau, the Agriculture and Rural Affairs Bureau, the Forestry and Grassland Bureau, the Emergency Management Bureau, the Public Complaints and Proposals Administration, the Natural Resources Bureau, the Ecological and Environmental Bureau, the Audit Office, the Finance Bureau, consulting agencies, universities, and other institutions. A total of 30 experts were invited to the symposium, with 15 experts from government departments (50%), 7 from consulting agencies (23.33%), and 8 from universities (26.67%). We obtained 30 completed expert questionnaires in the symposiums, the response rate was 100%.

Second, a typical survey was used to select 72 representative residents for face-to-face interviews according to the degree of impact, to listen to their specific views on DPM and demands for related interests. Specifically, firstly, a list of groups affected by DPM was obtained from government departments and categorized by occupation. Secondly, these residents were ranked according to their occupation and degree of disaster impact, with priority given to those who were most affected. Finally, the sample size of different occupational residents was determined according to the population proportion in each occupation and rounded up. The 72 representative residents included 25 farmers, 18 enterprise employees, 6 representatives of individual businesses, 4 representatives of local enterprises, 12 grassroot government officials, 5 retired workers, and 2 imams.

### 3.3. Identification of Social Stability Risks

Risk identification is a prerequisite for conducting social stability risk analysis. The main risk identification techniques included a literature review, brainstorming, flowcharting, discussions, and interviews [72]. The information from face-to-face interviews, which were conducted in July 2020, County D with 72 residents, and related literature [33,47] was combined to identify social stability risk factors for disaster-preventive migration in County D, which included 5 first-level risk factors and 14 s-level risk factors (Table 1).

### 3.4. Fuzzy Comprehensive Evaluation Model

Fuzzy comprehensive evaluation (FCE) is a method for comprehensive evaluation in a multi-criteria fuzzy decision environment using fuzzy sets [73]. The basic idea was to obtain the overall risk assessment results by constructing a mathematical model that comprehensively considered the level of influence of all risk factors. The method combined qualitative and quantitative approaches and enhanced the scientific validity of the SSRA. The FCE was divided into six steps, as follows.

In the first step, a set of influencing factors for the evaluation object was created. The influence factor set consisted of factors that affected the evaluation object. The influence factor set was denoted as:

(1) $$U=\left\{u_{1},u_{2},\cdots ,u_{i},\cdots ,u_{n}\right\}$$

(2) $$u_{i}=\left\{u_{i1},u_{i2},\cdots ,u_{ij},\cdots ,u_{ik}\right\}$$

where U is the set of influencing factors, *n* is the number of first-level risk factors included in U, $u_{i}\;(i=1,2,\cdots ,n)$ is the *i*th first-level risk factor in U, $u_{ij\;}(j=1,2,\cdots ,n)$ is the jth second-level factor in $u_{i}$, and k is the number of second-level risk factors.

The second step was to construct an evaluation set. The evaluation set included the evaluation values assigned to a certain evaluation indicator of the object. The evaluation set was expressed as:

(3) $$V=\left\{v_{1},v_{2},\cdots ,v_{m}\right\}=\left\{1,2,\cdots ,m\right\}$$

Here, V is the risk assessment set, $v_{1}$, $v_{2}$, ⋯, $v_{m}$ represent the risk level, and its corresponding rating values are 1, 2, ⋯, *m*.

The third step was to determine the weight assignment set. This information is often missing in comprehensive multi-indicator evaluations; the weight of each indicator should be determined based on expert opinions and knowledge. In this study, the frequency count [32] was used to determine the weights of first-level and second-level risk factors. Take the first-level risk factor set as an example:

(1) A weighting scheme was proposed by T (T>30) experts for each of the factors $u_{i}$ (i=1, 2,⋯,n) in the influencing factor set U.
(2) For the weights $a_{it}$ (t=1, 2,⋯,T) for factor $u_{i}$ provided by *T* experts, the maximum weight $G_{i}$ and the minimum weight $g_{i}$ were identified.
(3) An appropriate positive integer p was chosen such that $d=\frac{G_{i}-g_{i}}{p}$. The weights $a_{it}$ (t=1, 2,⋯,T) were divided into p groups, from the largest to the smallest, according to the group distance *d*.
(4) The frequencies or rates of the weights that fell within each group were calculated, and the group median ${\bar{a}}_{i}$ of the group with the maximum number of frequencies or rates was taken as the weight of factor $u_{i}$. Then, ${\bar{a}}_{i}$ was normalized to obtain the weight vector:
  (4) $$A=\left\{A_{1},A_{2},\cdots ,A_{n}\right\}=\left\{\frac{{\bar{a}}_{1}}{\sum_{i=1}^{n}{\bar{a}}_{i}},\frac{{\bar{a}}_{2}}{\sum_{i=1}^{n}{\bar{a}}_{i}},\cdots ,\frac{{\bar{a}}_{n}}{\sum_{i=1}^{n}{\bar{a}}_{i}}\right\}$$

Similarly, for the weights $a_{ijt}$ (t=1, 2,⋯,T) provided by T experts for the influencing factors of $u_{ij}\left(j=1,2,\cdots ,k\right)$ in $u_{i}$, the group median ${\bar{a}}_{ij}$ of the group with the maximum frequencies or rates, as obtained in the preceding steps, was regarded as the weight for factor $u_{ij}$. The weight vector of second-level risk factors was obtained after the normalization of ${\bar{a}}_{ij}$.

(5) $$A_{i}^{\prime }=\left\{A_{i1}^{\prime },A_{i2}^{\prime },\cdots ,A_{ik}^{\prime }\right\}=\left\{\frac{{\bar{a}}_{i1}}{\sum_{j=1}^{k}{\bar{a}}_{ij}},\frac{{\bar{a}}_{i1}}{\sum_{j=1}^{k}{\bar{a}}_{ij}},\cdots ,\frac{{\bar{a}}_{i1}}{\sum_{j=1}^{k}{\bar{a}}_{ij}}\right\}$$

In the fourth step, the fuzzy judgement matrices R and $R_{i}$ of U and $u_{i}$, respectively, were determined. In this study, the fuzzy judgement matrices were determined using fuzzy assessment methods based on the experts’ weights [74] by considering differences in the education level, cultural background, and familiarity with the assessment items of the experts. For the second-level risk factors, the weights of *T* experts were assumed to be $w_{1},w_{2},\cdots ,w_{T}$ and $\;w_{1}+w_{2}+\cdots +w_{T}=1$. The weight of one expert evaluating factor $u_{ij}$ as $v_{h}$ was denoted as $w_{t}^{\left(ijh\right)}$; then, the weight of the factor $u_{ij}$ being evaluated as $v_{h}$ (h=1, 2,⋯,m) was: $\overset{T}{\underset{t=1}{\sum }}w_{t}^{\left(ijh\right)}$. The membership of the evaluation factors $u_{ij}$ to the evaluation result $v_{h}$ in the evaluation set V was $r_{ijh}=\overset{T}{\underset{t=1}{\sum }}w_{t}^{\left(ijh\right)}/\overset{T}{\underset{t=1}{\sum }}w_{t}$, where $\overset{T}{\underset{t=1}{\sum }}w_{t}$ was the sum of all expert weights. After calculating the memberships of all factors of $u_{ij}$ using the aforementioned method, the fuzzy judgement array $R_{i}$ of the second-level risk factors was obtained, as follows:

(6) $$R_{i}={\left(r_{ijh}\right)}_{k\times m}=\left[\begin{array}{cccc} r_{i11} & r_{i12} & \cdots & r_{i1m}\\ r_{i21} & r_{i22} & \cdots & r_{i2m}\\ \cdots & \cdots & \cdots & \cdots \\ r_{ik1} & r_{ik2} & \cdots & r_{ikm} \end{array}\right]$$

R was obtained through calculating $R_{i}$, namely, let $B_{i}=A_{i}^{\prime }\cdot R_{i}$, then $R={\left[B_{1},B_{2},\cdots ,B_{n}\right]}^{T}$.

In the fifth step, FCE was performed. The first level of FCE was performed among the lowest layer of factors, while the second level of FCE was performed among the higher layers. The FCE vector of the first level was denoted as $B_{i}$, and that of the second level was denoted as B. The specific calculation process is shown in Equations (7) and (8).

(7) $$B_{i}=A_{i}^{\prime }\cdot R_{i}$$

(8) B=A·R

The fuzzy comprehensive matrices of different levels of evaluation factors were calculated according to Equations (7) and (8), and the risk levels were determined based on the maximum membership values [75].

The sixth step was to calculate the specific risk values. The second-level risk values were denoted as Z, and the first-level risk values were denoted as $Z_{i}^{\prime }$. The specific calculation process is shown in Equations (9) and (10).

(9) $$Z=B\times V^{T}$$

(10) $$Z_{i}^{\prime }=B_{i}\times V^{T}$$

The first- and second-level risk values were obtained through Equations (9) and (10) to provide the basis for risk management decisions. Social stability risks level and reference standard are shown in Table 2.

## 4. Results

The social stability risks of disaster-preventive migration were assessed according to the FCE steps described earlier.

(1) The influencing factor set of the evaluation object was established. The influencing factor set of an evaluation object was U”=“ {social stability risk of DPM}”={“u_1”,“u_2”,“u_3”,“u_4”,“u_5”}={“compensation risk, cultural risk, livelihood recovery risk, geological disaster risk,  public opinion risk”}” , $u_{i}=\left\{u_{i1},u_{i2},\ldots ,u_{ik}\right\}i=1,\;2,\ldots ,5$.
(2) The evaluation set was $V=\left\{v_{1},v_{2},v_{3},v_{4},v_{5},\right\}=\left\{1,2,3,4,5\right\}$. These five levels represented the five possible evaluation results, defined as follows: very low risk level, low risk level, medium risk level, high risk level, and very high-risk level.
(3) Taking p=6, the weight vectors of second-level risk factors and first-level risk factors were obtained:
  $$A_{1}^{\prime }=\left\{A_{11}^{\prime },A_{12}^{\prime },A_{13}^{\prime }\right\}=\left\{0.308,0.333,0.359\right\}$$
  $$A_{2}^{\prime }=\left\{A_{21}^{\prime },A_{22}^{\prime },A_{23}^{\prime }\right\}=\left\{0.363,0.392,0.245\right\}Z_{i}^{\prime }=B_{i}\times V^{T}$$
  $$A_{3}^{\prime }=\left\{A_{31}^{\prime },A_{32}^{\prime },A_{33}^{\prime }\right\}=\left\{0.449,0.187,0.364\right\}$$
  $$A_{4}^{\prime }=\left\{A_{41}^{\prime },A_{42}^{\prime }\right\}=\left\{0.530,0.470\right\}$$
  $$A_{5}^{\prime }=\left\{A_{51}^{\prime },A_{52}^{\prime },A_{53}^{\prime }\right\}=\left\{0.508,0.209,0.283\right\}$$
  $$A=\left\{A_{1},A_{2},A_{3},A_{4},A_{5}\right\}=\left\{0.407,0.116,0.233,0.093,0.151\right\}$$
(4) The expert weights were determined through the questionnaires administered to the experts, based on which the fuzzy judgement matrices $R_{i}$ and R of the first- and second-level indicators were established using an FCE method:
  $$R_{1}=\left\{\begin{array}{ccccc} 0.119 & 0.151 & 0.175 & 0.325 & 0.230\\ 0.095 & 0.127 & 0.278 & 0.294 & 0.206\\ 0.159 & 0.175 & 0.206 & 0.214 & 0.246 \end{array}\right\}$$
  $$R_{2}=\left\{\begin{array}{ccccc} 0.135 & 0.167 & 0.357 & 0.167 & 0.175\\ 0.294 & 0.349 & 0.151 & 0.095 & 0.111\\ 0.151 & 0.119 & 0.373 & 0.159 & 0.198 \end{array}\right\}$$
  $$R_{3}=\left\{\begin{array}{ccccc} 0.056 & 0.127 & 0.087 & 0.468 & 0.262\\ 0.278 & 0.317 & 0.159 & 0.111 & 0.135\\ 0.317 & 0.341 & 0.135 & 0.143 & 0.063 \end{array}\right\}$$
  $$R_{4}=\left\{\begin{array}{ccccc} 0.278 & 0.214 & 0.262 & 0.183 & 0.063\\ 0.127 & 0.540 & 0.127 & 0.135 & 0.071 \end{array}\right\}$$
  $$R_{5}=\left\{\begin{array}{ccccc} 0.056 & 0.111 & 0.183 & 0.397 & 0.254\\ 0.032 & 0.119 & 0.278 & 0.365 & 0.206\\ 0.063 & 0.151 & 0.294 & 0.270 & 0.222 \end{array}\right\}$$
  $$R=\left\{\begin{array}{ccccc} 0.125 & 0.151 & 0.220 & 0.275 & 0.228\\ 0.201 & 0.227 & 0.280 & 0.137 & 0.156\\ 0.193 & 0.241 & 0.118 & 0.283 & 0.166\\ 0.207 & 0.367 & 0.198 & 0.160 & 0.067\\ 0.053 & 0.124 & 0.234 & 0.354 & 0.235 \end{array}\right\}$$
(5) The indicator system of the social stability risks of disaster-preventive migration was divided into two levels. FCE was performed first for second-level indicators and then for first-level indicators.

The FCE results of social stability risks of disaster-preventive migration were obtained as follows:

$$B_{1}=A_{1}^{\prime }\cdot R_{1}=\left[0.308,0.333,0.359\right]\cdot \left[\begin{array}{ccccc} 0.119 & 0.151 & 0.175 & 0.325 & 0.230\\ 0.095 & 0.127 & 0.278 & 0.294 & 0.206\\ 0.159 & 0.175 & 0.206 & 0.214 & 0.246 \end{array}\right]=\left[0.125,0.151,0.220,0.275,0.228\right]$$

$$B_{2}=A_{2}^{\prime }\cdot R_{2}=\left[0.363,0.392,0.245\right]\cdot \left[\begin{array}{ccccc} 0.135 & 0.167 & 0.357 & 0.167 & 0.175\\ 0.294 & 0.349 & 0.151 & 0.095 & 0.111\\ 0.151 & 0.119 & 0.373 & 0.159 & 0.198 \end{array}\right]=\left[0.201,0.227,0.280,0.137,0.156\;\right]$$

$$B_{3}=A_{3}^{\prime }\cdot R_{3}=\left[0.449,0.187,0.364\right]\cdot \left[\begin{array}{ccccc} 0.056 & 0.127 & 0.087 & 0.468 & 0.262\\ 0.278 & 0.317 & 0.159 & 0.111 & 0.135\\ 0.317 & 0.341 & 0.135 & 0.143 & 0.063 \end{array}\right]=\left[0.193,0.241,0.118,0.283,0.166\right]$$

$$B_{4}=A_{4}^{\prime }\cdot R_{4}=\left[0.530,0.470\right]\cdot \left[\begin{array}{ccccc} 0.278 & 0.214 & 0.262 & 0.183 & 0.063\\ 0.127 & 0.540 & 0.127 & 0.135 & 0.071 \end{array}\right]=\left[0.207,0.367,0.198,0.160,0.067\right]$$

$$B_{5}=A_{5}^{\prime }\cdot R_{5}=\left[0.508,\;0.209,\;0.283\right]\cdot \left[\begin{array}{ccccc} 0.056 & 0.111 & 0.183 & 0.397 & 0.254\\ 0.032 & 0.119 & 0.278 & 0.365 & 0.206\\ 0.063 & 0.151 & 0.294 & 0.270 & 0.222 \end{array}\right]=\left[0.053,0.124,0.234,0.354,0.235\right]$$

where $B_{1}$ indicated compensation risks, $B_{2}$ cultural risks, $B_{3}$ livelihood restoration risks, $B_{4}$ geohazard risks, and $B_{5}$ public opinion risks.

The following was obtained based on the results of the preceding evaluation:

$$R={\left[B_{1},B_{2},\cdots ,B_{n}\right]}^{T}=\left[\begin{array}{ccccc} 0.125 & 0.151 & 0.220 & 0.275 & 0.228\\ 0.201 & 0.227 & 0.280 & 0.137 & 0.156\\ 0.193 & 0.241 & 0.118 & 0.283 & 0.166\\ 0.207 & 0.367 & 0.198 & 0.160 & 0.067\\ 0.053 & 0.124 & 0.234 & 0.354 & 0.235 \end{array}\right]$$

A comprehensive evaluation based on Equation (8) yielded the following comprehensive evaluation vector:

$$B=A\cdot R=\left[0.407,0.116,0.233,0.093,0.151\right]\cdot \left[\begin{array}{ccccc} 0.125 & 0.151 & 0.220 & 0.275 & 0.228\\ 0.201 & 0.227 & 0.280 & 0.137 & 0.156\\ 0.193 & 0.241 & 0.118 & 0.283 & 0.166\\ 0.207 & 0.367 & 0.198 & 0.160 & 0.067\\ 0.053 & 0.124 & 0.234 & 0.354 & 0.235 \end{array}\right]=\left[0.146,0.197,0.204,0.262,0.191\right]$$

(6) The SSRA value for disaster-preventive migration and the first-level risk factor values for social stability $Z_{i}^{\prime }$ in disaster-preventive migration were calculated based on Equations (9) and (10):
  $$Z=B\times V^{T}=\left[0.146,0.197,0.204,0.262,0.191\right]\times \left[1,2,3,4,5\right]=3.155$$
  $$Z_{1}^{\prime }=B_{1}\times V^{T}=\left[0.125,0.151,0.220,0.275,0.228\right]\times \left[1,2,3,4,5\right]=3.327$$
  $$Z_{2}^{\prime }=B_{2}\times V^{T}=\left[0.201,0.227,0.280,0.137,0.156\right]\times \left[1,2,3,4,5\right]=2.819$$
  $$Z_{3}^{\prime }=B_{3}\times V^{T}=\left[0.193,0.241,0.118,0.283,0.166\right]\times \left[1,2,3,4,5\right]=2.989$$
  $$Z_{4}^{\prime }=B_{4}\times V^{T}=\left[0.207,0.367,0.198,0.160,0.067\right]\times \left[1,2,3,4,5\right]=2.514$$
  $$Z_{5}^{\prime }=B_{5}\times V^{T}=\left[0.053,0.124,0.234,0.354,0.235\right]\times \left[1,2,3,4,5\right]=3.595$$
  where $Z_{1}^{\prime }$ is the assessment value of compensation risks, $Z_{2}^{\prime }$ is the assessment value of cultural risks, $Z_{3}^{\prime }$ is the assessment value of livelihood recovery risks, $Z_{4}^{\prime }$ is the assessment value of geological hazard risks, and $Z_{5}^{\prime }$ is the assessment value of public opinion risks.

**Figure 2:**
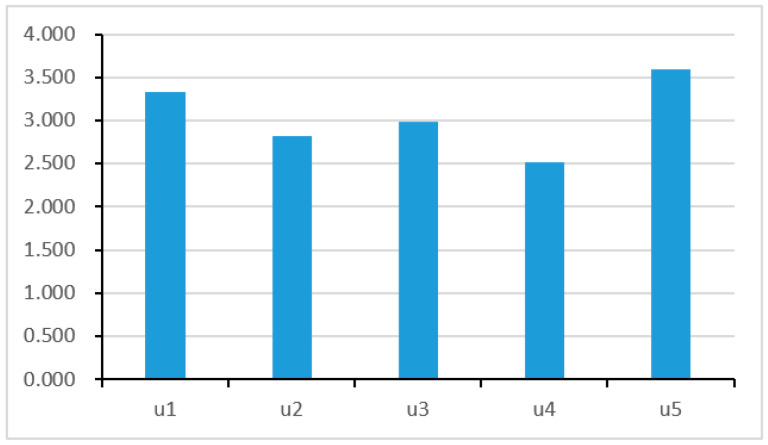
Social stability risk level of first-level risk factors.

The results of the comprehensive SSRA showed that the overall social stability risk of disaster-preventive migration in County D is ‘high’. Specifically, compensation risks and public opinion risks were classified as ‘high’, while cultural risks, livelihood restoration risks, and geological hazard risks were classified as ‘medium’. Figure 2 shows the scores of first-level risk factors for social stability in the risk assessment of disaster-preventive migration in County D, ranked in descending order: Public opinion risks ($u_{5}$) > compensation risks ($u_{1}$) > livelihood recovery risks ($u_{3}$) > cultural risks ($u_{2}$) > geological hazard risks ($u_{4}$).

## 5. Discussion

The results showed a ‘high’ overall social stability risk of disaster-preventive migration in County D, which may lead to social instability if risk prevention measures are not taken. The risk values of public opinion risks and compensation risks were the highest among the first-level risk factors, and geological disaster risks have a major influence on the willingness of DPM. Thus, to control the social stability risks of DPM in County D, decision-makers should primarily consider these three major risk factors.

(1) Public opinion risks: This risk showed three aspects, namely, the level of openness and transparency of information on disaster-preventive migration, the level of public participation, and the government’s response to public opinions. Information plays an important role in risk management and is fundamental to risk communication [76]. The open and transparent flow of public information on disaster-preventive migration between the government and the society has become one of the important factors affecting the validity of risk governance [77]. Transparent information on disaster-preventive migration can help reduce public fears and avoid misunderstandings among affected people and can enable them to trust disaster-preventive resettlement projects [78,79]. Public opinion risks stemmed from a lack of information transparency. Moreover, the government’s suppression, minimization, non-response, or inappropriate response to public opinion would aggravate public opinion and may have significant impacts.

Voluntariness and prior informed consent are important principles in the relocation and resettlement of disaster-affected migrants, and public participation is key for the implementation of these principles [80]. The central government, local governments, as well as several stakeholders and actors were involved in disaster-preventive migration in County D, with different stakeholders and actors having different interests and motivations in the project [81]. The World Bank has pointed out the importance of timely consultation with stakeholders on resettlement programs and of providing them with opportunities to participate in the planning, implementation, and monitoring of resettlement [82]. Furthermore, studies have shown that inadequate public participation in disaster-preventive migration may lead to conflicts among the stakeholders [83].

In fact, the government of County D had proposed a plan for relocation as early as 2003 and had conducted preliminary research on several proposed relocation sites. However, the county relocation plan was placed on hold. For a decade or so, the government failed to disclose information on the rationale for the relocation and the mode of relocation, which resulted in a widespread negative public opinion. Moreover, the government’s evasive responses to such public opinion resulted in inadvertent harm to the residents. For example, some residents wanted to build new houses to improve their living conditions, but the overwhelming negative public opinion on county relocation prevented them from doing so. In 2019, the Ministry of Natural Resources dispatched a ‘Letter of Recommendations on the Prevention and Control of Geological Disasters in County D, Diqing Prefecture, Yunnan Province’ to the General Office of the Yunnan Provincial People’s Government; this letter clearly stated that ‘County D has become one of the counties most seriously threatened by geological disasters in Yunnan province and even in the country. The current development of the county exceeds its urban environmental capacity, posing severe potential risk from geological disasters to people’s lives and properties’, thereby bringing the relocation of County D back on the agenda. However, relevant information has remained ‘confidential’, and there was a lack of public participation in the formulation of the county’s relocation plan, which led to the risks of public opinion being ranked first among all first-level risk factors. It is recommended that the People’s Government of County D should seek instructions from the relevant departments at higher levels as soon as possible to clarify the policies and measures involved in relocation and to disclose the information to the public in a timely manner.

(2) Compensation risks: This risk was reflected in the compensation for houses and attached buildings, land and its appurtenances, and disaster-preventive resettlement of migrants. Studies have shown that the relocation compensation program is a core factor influencing the willingness of people to relocate and is also a key factor in reducing social stability risks [33,84,85]. County D is a multi-ethnic area where Tibetans are the main ethnic group; the residents’ houses consist of wooden houses, brick houses, and cement houses. With the development of the tourism economy in County D, housing rental income has become an important source of income for the residents in the area. Therefore, residents had high expectations from relocation housing subsidies. Residents hoped to receive additional compensation for relocation in ethnic minority areas on top of the national compensation standard.

County D is rich in forestry resources, and the main forestry products are cordyceps and pine mushrooms. Therefore, farmers in Town S of County D currently have higher income levels than farmers in other parts of the county. Compensation for the forest land and its associated above-ground appurtenances in the relocation was one of the main concerns of farmers. Although the government promised that the mountain and forestry rights of S town would remain after the relocation and that residents could still return to S town to collect matsutake, cordyceps, and other forest products, the residents said that they basically would not come back to collect the forest products because the planned relocation site is too far from S town, and the cost of coming back to collect the forest products is too high. In addition, they stated that if they do not live there, the forest products, such as matsutake and cordyceps in the contracted forest land, would be collected by other people, and new conflicts and disputes would arise. These factors have led the residents to increase their compensation expectations.

In the implementation of resettlement, the provided compensation included cash, land, and employment [31]. Cash compensation is the simplest form of compensation and is used often in resettlement. However, cash compensation is not always effective. Some relocatees were not good at managing cash, and some used resettlement subsidies for weddings or gambling, which prevented them from relocating [86]. S town is a Tibetan settlement. Tibetan residents have the tradition of living in compact family communities, with several generations living together and building a single Tibetan house with front and back yards and livestock sheds, among other things. They are unable to accept apartments. The demand for resettlement homesteads became a priority during relocation, surpassing the demand for cash and employment compensation. Therefore, the relocation compensation scheme should fully consider the demands of different types of people, respect the customs of ethnic minorities, and meet the specified cultural requirements of ethnic minorities.

(3) Geological disaster risks: This risk is measured in terms of the “likelihood of occurrence” and the “magnitude of damage caused by geological hazards”, which correspond to the “likelihood” and “threat” dimensions of risk perception, respectively [87,88,89]. Therefore, the geological disaster risks in this paper is, in fact, geological disaster risk perception. Some studies have found that disaster risk perception has a significant impact on DPM willingness [90]. Different measures of risk perception have inconsistent effects on migration decisions [91]. People’s willingness to relocate will increase when the potential of a disaster is higher and the threat is greater [92]. The willingness of DPM has a direct impact on the social stability risk. Forced migration is more likely to cause social instability than voluntary migration [93,94]. The geological disaster risk is an inverse indicator, and its risk value is the lowest among the five primary risk factors, which suggests that experts had a higher risks perception of geological disaster in County D. However, during interviews with residents, we found that most residents’ risk perception of geological disaster in County D was opposite to that of the experts. They believe that although minor geological disasters were once very common there, there had never been a serious geological disaster. They appeared very sure that disasters would not happen in the future, because the local government has already invested a lot of manpower and resources in the prevention and control of geological disasters.

Expert versus nonexpert (local resident) differences in geological disaster risk perception will cause great obstacles to the implementation of DPM. To make the DPM project in County D proceed smoothly, the People’s Government of County D should resolve the potential differences in geological disaster risk perception between experts and nonexperts (aboriginal) as quickly as possible by highlighting the potential severity of geological disasters, their effects on people’s lives and safety, and the restricted future development of the county, among other points.

## 6. Conclusions

Southwestern China is an area prone to geological disasters. To reduce the risk of disasters, many disaster-preventive relocation and resettlement programs have been implemented. Although disaster-preventive migration is an important method for disaster risk management, migration itself entails potential social stability risks, which would affect the harmony and stability of the society if not implemented properly [95]. How to identify and evaluate the social stability risk of DPM in County D is the core issue of this paper. In order to make the conclusions scientific and reasonable, this study identified the social stability risk of DPM in County D based on feedback from symposiums, in-depth interviews, and literature and assessed it by a fuzzy comprehensive evaluation method considering multiple experts’ weights. The following conclusions were obtained from the research:

(1) The social stability risk of DPM in County D includes five first-level risk factors, which are public opinion risks, compensation risks, livelihood recovery risks, cultural risks, geological disaster risks.
(2) The overall social stability risk level of DPM in County D is ‘high’. In terms of importance, the five first-level risk factors were ranked as follows: public opinion risks > compensation risks > livelihood recovery risks > cultural risks > geological disaster risks. Expert versus nonexpert differences in geological disaster risk perception will cause great obstacles to the implementation of DPM.

## Figures and Tables

**Table 1:** Social stability risk factors for disaster-preventive migration.

Target Layer	First-Level Risk Factors	Second-Level Risk Factors
Social stability risk factors of DPM	Compensation risks	Housing compensation
		Compensation for land acquisition
		Settlement allowance
	Cultural risks	Inability to adapt to the lifestyle
		Integration of ethnicities
		Changes in social networks
	Livelihood recovery risks	Loss of forest and land resources
		Job opportunities and income issues
		Inability to meet expectations of living environment
	Risk of geological hazards	Possibility of geological hazards
		Magnitude of damage caused by geological hazards
	Risks linked to public opinion	Level of openness and transparency of information
		Level of public participation
		Government response to public opinion

**Table 2:** Social stability risks’ level and reference standard.

Social Stability Risks	Very Low	Low	Medium	High Risk	Very High
Risk value (Z)	(0, 1]	(1, 2]	(2, 3]	(3, 4]	(4, 5]
Grade	1	2	3	4	5

## Data Availability

Data sharing not applicable.
